# Totally endoscopic aortic valve replacement with concomitant septal myectomy

**DOI:** 10.1093/icvts/ivaf030

**Published:** 2025-02-18

**Authors:** Sahri Kim, Jae Suk Yoo

**Affiliations:** Department of Thoracic and Cardiovascular Surgery, Asan Medical Center, University of Ulsan College of Medicine, Seoul, Korea; Department of Thoracic and Cardiovascular Surgery, Asan Medical Center, University of Ulsan College of Medicine, Seoul, Korea

**Keywords:** minimally invasive surgery, aortic valve replacement, septal myectomy

## Abstract

Left ventricular outflow tract obstruction may become apparent after successful aortic valve replacement for severe aortic stenosis, necessitating both aortic valve replacement and septal myectomy. Despite significant advancements in minimally invasive surgery, the current literature on concurrent minimally invasive cardiac procedures is limited due to the varying optimal exposures required for each procedure. In this context, we present a case of aortic valve replacement with concomitant septal myectomy using a minimally invasive, totally endoscopic approach.

## CASE PRESENTATION

A 63-year-old woman with no significant underlying disease was diagnosed with aortic valve calcification during routine computed tomography (CT) performed as part of a medical checkup. The patient presented with New York Heart Association class II symptoms and was referred for further evaluation. Transthoracic and transoesophageal echocardiography revealed severe aortic stenosis with mild aortic and mitral regurgitation. No interventricular septum abnormality was detected in these initial studies; however, cardiac CT revealed basal septal hypertrophy of the left ventricular wall. Despite this, the left ventricular outflow tract (LVOT) pressure gradient was 3 mmHg, and an aortic valve replacement (AVR) with concomitant septal myectomy was planned to prevent LVOT obstruction (LVOTO).

**Figure 1:**
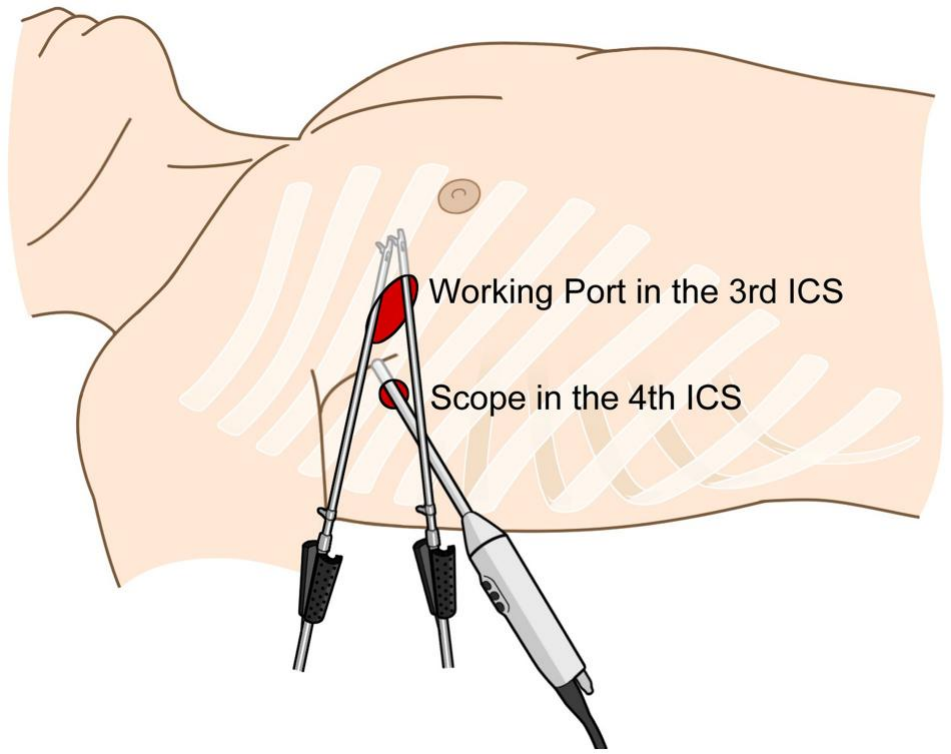
Schematic image showing the main thoracotomy (third intercostal space, midclavicular line) and port insertion site (fourth intercostal space, anterior axillary line) for the two-port totally endoscopic approach.

Under cardiopulmonary bypass, a thoracotomy of 2–3 cm was performed in the third intercostal space along the right midclavicular line, and a 10-mm trocar was inserted in the fourth intercostal space at the anterior axillary line. An additional soft tissue retractor was applied to the main thoracotomy incision (Fig. 1). An aortotomy was performed, and the aortic valve leaflets were resected. After placing annular stitches on the right coronary cusp side for septal exposure, septal myectomy was performed. The annular stitches were then completed, followed by the insertion of a 21-mm Inspiris aortic valve. The aortotomy was closed, and a root cannula was inserted for de-airing before the aortic clamp was released (Video 1). Cardiopulmonary bypass was gradually weaned, and drain catheters were inserted into the pericardium and pleura.

**Figure 2:**
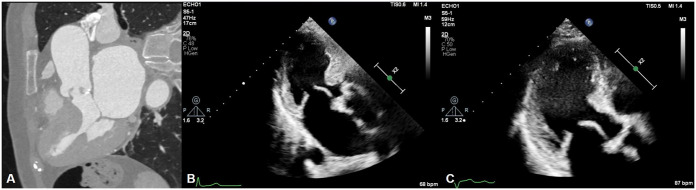
Comparison between (**A**) preoperative cardiac computed tomography, (**B**) pre- and (**C**) postoperative echocardiography (parasternal long axis view).

The operation lasted 210 min, with a total pump time of 130 min and an aortic cross-clamp time of 94 min. The patient remained in the intensive care unit for 26 h. Transthoracic echocardiography performed on postoperative Day 5 revealed good prosthetic valve function with no paravalvular leakage. Although mild midventricular hypertrophy persisted, no LVOTO or systolic anterior motion of the mitral valve was observed, and the LVOT pressure gradient was 4 mmHg (Fig. 2). The patient was discharged on postoperative Day 5 without complications.

## DISCUSSION

LVOTO following AVR in severe aortic stenosis is a relatively under-recognized phenomenon, partly because it can remain undetected in the presence of a fixed obstruction, such as aortic stenosis, which causes high end-systolic pressure. LVOTO may become apparent after successful AVR, which relieves the pressure that previously held the ventricular walls apart and masked basal septal hypertrophy or the possibility of septal anterior motion [1]. Kayalar *et al.* [2] assessed the role and safety of concomitant septal myectomy during AVR for severe aortic stenosis, and in 45% of their study group, neither preoperative nor intraoperative echocardiography suggested prominent septal hypertrophy, which could cause LVOTO. Therefore, as demonstrated in our case, a thorough examination using other modalities, such as CT, is crucial to identify patients at high risk for LVOTO. Postoperative CT and echocardiography are also recommended to confirm whether the anticipated outcomes have been achieved.

In recent years, the concept of ‘suicide left ventricle’ after transcatheter aortic valve implantation has emerged, highlighting the risk of LVOTO development due to the relief of afterload in patients with basal septal hypertrophy masked by sever aortic stenosis [3]. For such patients, totally endoscopic AVR combined with septal myectomy represents a promising alternative, addressing both immediate postoperative outcomes and long-term durability by eliminating LVOTO.

Right anterior thoracotomy was our preferred approach when only AVR was planned. However, when a concomitant myectomy is needed, a median sternotomy is usually performed because additional manoeuvres, such as pressing the RV with a sponge stick, are needed for better visualization [2, 4, 5]. Nevertheless, we successfully performed AVR and septal myectomy using a fully endoscopic approach via a right mini-thoracotomy, achieving optimal visualization.

Our centre employs a two-port system for totally endoscopic surgery, strategically positioning the camera port one intercostal level lower than the main thoracotomy incision, placing the scope on the operator’s right side. This contrasts with the more commonly used approach where the scope is positioned on the left side. Our setup prevents collisions between the left-hand instrument and the scope without requiring an additional port incision and ensures the scope does not interfere with the surgical field or collide with the operator’s body. By placing the camera in the fourth intercostal space and utilizing a 30-degree scope, this approach provides excellent visualization while minimizing the risk of bleeding and surgical complications.

This minimally invasive technique required total pump and aortic cross-clamping times comparable to those of the conventional sternotomy method, providing excellent exposure and a smaller incision, which are beneficial to the patient. To our knowledge, although individual thoracoscopic approaches for AVR and myectomy have been reported, a totally endoscopic approach for simultaneous AVR and septal myectomy, particularly using a two-port system, has not been previously reported. Thus, this report marks an advancement in surgical approaches.

## Data Availability

The data underlying this article are available within the article.
